# Gender of offspring and long-term maternal breast cancer risk

**DOI:** 10.1054/bjoc.1999.1044

**Published:** 2000-02-01

**Authors:** J Wohlfahrt, M Melbye

**Affiliations:** Department of Epidemiology Research, Danish Epidemiology Science Centre, Statens Serum Institut, Artillerivej 5, Copenhagen, DK-2300, Denmark

**Keywords:** gender, pregnancy, breast cancer incidence, population-based cohort study

## Abstract

Gender of offspring is influenced by maternal hormonal level during pregnancy, which is blieved to influence the subsequent maternal breast cancer risk. However, analysing national birth and cancer registrations in a cohort of 998 499 women, we found no association between gender of offspring and subsequent breast cancer risk. © 2000 Cancer Research Campaign


*British Journal of Cancer* (2000) **82**(5), 1070–1072
© 2000 Cancer Research Campaign
DOI: 10.1054/ bjoc.1999.1044, available online at http://www.idealibrary.com on IDEAL®

Short Communication

### Gender of offspring and long-term maternal breast cancer risk

**J Wohlfahrt and M Melbye**

Department of Epidemiology Research, Danish Epidemiology Science Centre, Statens Serum Institut, Artillerivej 5, DK-2300 Copenhagen S, Denmark

**Summary** Gender of offspring is influenced by maternal hormonal level during pregnancy, which is blieved to influence the subsequent maternal breast cancer risk. However, analysing national birth and cancer registrations in a cohort of 998 499 women, we found no association between gender of offspring and subsequent breast cancer risk. © 2000 Cancer Research Campaign

**Keywords:** gender; pregnancy; breast cancer incidence; population-based cohort study

Childbirth induces a short-term increase and a long-term decrease in a mother's breast cancer risk (Lambe et al, 1994; Albrektsen et al, 1995*a*). Hormonal levels during pregnancy may influence both effects. This has been investigated by looking at the maternal breast cancer risk following a pregnancy with characteristics associated with elevated hormonal levels (Enger et al, 1997; Troisi et al, 1998; Wohlfahrt and Melbye, 1999). The maternal breast cancer risk according to gender distribution of offspring has for the same reason attracted interest since gender differences in the maternal level of serum alfa-fetoprotein, human chorionic gonadotrophin (hCG) and sex hormone-binding globulin have been reported that might also be related to maternal breast cancer risk (reviewed in Hsieh et al, 1999). A large Norwegian cohort study with 3937 cases found no association between breast cancer risk and gender distribution of offspring (Albrektsen et al, 1995*b*), but recently a Swedish case–control study including 2328 cases found that deliveries of male offspring had a protective effect (Hsieh et al, 1999). The mechanisms behind the hormonal influence on the short-term increase and long-term decrease in breast cancer risk following childbirth are believed to be different (Adami et al, 1998), and it is therefore important to investigate the effects separately. In a recent, large cohort study including 9495 cases we have found no modification by gender of offspring of the short-term effect (Wohlfahrt and Melbye, 1999). In the present study we investigated whether this is also true for the long-term effect of childbirth.

### MATERIALS AND METHODS

Since 1 April 1968, the Civil Registration System (CRS) in Denmark has assigned an individually unique national registration number to all citizens. This number permits accurate linkage of information from different registries. The Civil Registration System also keeps updated information on gender and dates of live births, emigration and vital status. Information on still-births was available from the National Birth Register. To identify multiple deliveries we looked for children born to the same mother within 2 days. A research parity database was established from the CRS including all women born between 1 April 1935 and 31 March 1978, as earlier described (Melbye et al, 1997; Westergaard et al, 1997). Based on the person-identifiable CRS number, a linkage was performed with the Danish Breast Cancer Group's registry (DBCG) (Andersen and Mouridsen, 1988; Kroman et al, 1997) giving information on registered invasive breast cancers in the period from 1 January 1978 to 30 September 1994. The DBCG's registry was found to contain information on 94% of all breast cancer patients reported to the Danish Cancer Registry, which has nearly complete registration of all incident cases of malignant neoplasms diagnosed in Denmark since 1943 (Storm, 1991). The impact of gender on the incidence of breast cancer was investigated in a follow-up study analysed by log-linear Poisson regression models (Breslow and Day, 1987). All women entered the follow-up for breast cancer diagnoses on 1 January 1978, or on the date of their first childbirth, whichever came last. The period at risk continued until breast cancer, death, emigration or 30 September 1994, whichever occurred first. Incidence rate ratios are referred to as relative risks. In all analyses adjustment was made for age (< 30, 31, 32, … 57, 58), calendar period (1978–82, 1983–87, 1988–92, 1993–94) and age at first birth (< 20, 20–24, 25–29, 30–34, 35+). The gender of $n$ birth was categorized as ($n$ – 1 parous or less, boy, girl). The first parameter in the gender variables were redundant when the gender variables for all births were included and these parameters were therefore set to zero.

### RESULTS

In all we observed 9495 cases of breast cancer during 12.8 million years of follow-up. All the following analyses are restricted to 5 or more years after latest birth in mothers with a history of only single births in order to focus on long-term effects of single births. With this restriction we observed 8607 cases during 8.7 million years of follow-up. Using 10 years instead of 5 years gave similar results. The mother's risk of breast cancer decreased significantly with number of births: 1 birth: 1 (reference); 2 births: 1.0 (0.9–1.0);

*Received 14 May 1999*
*Revised 13 August 1999*
*Accepted 13 August 1999*

*Correspondence to:* M Melbye

**Table 1** Relative maternal risk[a] of breast cancer 5 or more years after latest birth according to gender distribution of offspring

| Number of girls | Number of boys | | | | | |
|---|---|---|---|---|---|---|
| | 0 | 1 | 2 | 3 | 4+ | All |
| 0 | – | 1.0 (0.9–1.1) | 1.0 (0.9–1.1) | 0.9 (0.8–1.1) | 0.6 (0.4–0.8) | 1.2 (1.1–1.2) |
| 1 | 1 (ref.) | 0.9 (0.9–1.0) | 0.9 (0.8–1.0) | 0.7 (0.6–0.9) | 0.5 (0.3–0.9) | 1 (ref.) |
| 2 | 1.0 (0.9–1.1) | 0.9 (0.8–0.9) | 0.8 (0.6–0.9) | 0.5 (0.4–0.8) | 0.2 (0.1–0.7) | 0.9 (0.8–1.0) |
| 3 | 0.8 (0.6–0.9) | 0.7 (0.6–0.9) | 0.7 (0.4–1.0) | 0.2 (0.1–0.9) | 0.9 (0.3–2.8) | 0.7 (0.7–0.8) |
| 4+ | 0.7 (0.4–0.1) | 0.4 (0.2–0.9) | 1.3 (0.7–2.4) | 0.4 (0.1–2.7) | – (no cases) | 0.7 (0.5–0.9) |
| All | 1.1 (1.1–1.2) | 1 (ref.) | 1.0 (0.9–1.0) | 0.9 (0.8–1.0) | 0.6 (0.4–0.7) | |

[a]All relative risks are adjusted for attained age, calendar period and age at first birth and with 95% confidence interval. The effects of number of boys and number of girls are furthermore mutually adjusted.

**Table 2** Relative maternal risk[a] of breast cancer 5 or more years after latest birth according to gender of offspring in 1st to 6th birth

| Offspring birth order | Gender of offspring | |
|---|---|---|
| | Boy | Girl |
| 1 | 1 (ref.) | 1.0 (1.0–1.0) |
| 2 | 1 (ref.) | 1.0 (0.9–1.0) |
| 3 | 1 (ref.) | 0.9 (0.9–1.0) |
| 4 | 1 (ref.) | 1.0 (0.9–1.2) |
| 5 | 1 (ref.) | 1.0 (0.7–1.6) |
| 6 | 1 (ref.) | 1.0 (0.4–2.7) |

[a]All relative risks are adjusted for attained age, calendar period, age at first birth and gender of 1st to 6th birth and with 95% confidence interval.

3 births: 0.9 (0.8–0.9); 4 births: 0.7 (0.6–0.8); 5+ births: 0.5 (0.4–0.7). Table 1 shows the risk of breast cancer according to the gender distribution of the mother's offspring. We observed that women with many compared to few boys, and women with many compared to few girls, had a lower breast cancer risk. However, the effects are similar and can be described more simply by the total number of births. This can be seen by the very similar estimates within the diagonals from left-bottom to right-top, i.e. within strata of similar parity (Table 1, not including the All category). Within the parity-specific strata the distribution of boys and girls does not modify the risk. The pattern was the same in women younger than 45 years of age and in women aged 45 years or older. In an alternative approach we estimated the gender difference in the long-term effects of the 1st to 6th birth (Table 2). The effect of 1st to 6th birth was not modified by the gender of the offspring.

### DISCUSSION

Our study shows that gender of offspring does not modify the effect of a childbirth on the breast cancer risk. This is true for both the short-term increase and the long-term decrease of breast cancer risk after a childbirth. The gender modification of the long-term effect was investigated by studying breast cancer risk 5 or more years after the latest birth according to the gender distribution of offspring as well as the effects of each birth. The long-term decrease in risk following a childbirth is believed to originate from permanent changes in the susceptibility of the stem cells, changes that perhaps partly are determined by the hormonal level during pregnancy (Adami et al, 1998). We therefore used these approaches as it is most plausible that a potential gender-induced modification of the long-term effect would be an effect of the gender of all previous births some years after the latest birth, i.e. after the most marked effects of these transient negative effects of the births.

The short-term effect of a childbirth is, on the other hand, believed to be due to hormonally induced growth of premalignant and malignant tumours. A study of the gender modification of the short-term effect should therefore either focus on the latest birth in a short time-interval after the latest birth or the short-term effects of each of the births separately. Studying the gender modification of the short-term effect according to gender distribution of all births disregarding the order of appearance and only restricting to young women, as in the study by Hsieh et al of the gender effect in 'childbearing ages' (Hsieh et al, 1999), is therefore most likely going to obscure the true short-term effect. As argued above, such an approach is more appropriate in the study of long-term effects. We have recently looked at the effect of gender of the most recent birth within the first 5 years following birth (Wohlfahrt and Melbye, 1999) and observed no modifying effect of gender of offspring. Based on these findings in a large population-based cohort study we conclude that gender of offspring modifies neither the short- nor the long-term effect of breast risk following childbirth. Our findings do not necessarily imply that the hormones related to gender are of no importance in the aetiology of breast cancer, but probably illustrate that the gender differences in hormonal levels during pregnancy are small compared with the hormonal changes induced by a pregnancy irrespective of gender.

### ACKNOWLEDGEMENTS

This study was supported by the US Army Breast Cancer Research Program (grant no. DAMD17–96–1–632) and the Danish National Research Foundation.

### REFERENCES

Adami H-O, Signorello LB and Trichopoulos D (1998) Towards an understanding of breast cancer etiology. *Semin Cancer Biol* **8:** 255–262

© 2000 Cancer Research Campaign

Albrektsen G, Heuch I and Kvåle G (1995*a*) The short-term and long-term effect of a pregnancy on breast cancer risk: a prospective study of 802 457 parous Norwegian women. *Br J Cancer* **72:** 480–484

Albrektsen G, Heuch I and Kvåle G (1995*b*) Multiple births, sex of children and subsequent breast cancer risk for the mothers: a prospective study in Norway. *Int J Cancer* **60:** 341–344

Andersen KW and Mouridsen HT (1988) Danish Breast Cancer Cooperative Group (DBCG): a description of the register of the nationwide program for primary breast cancer. *Acta Oncol* **27:** 627–643

Breslow NE and Day NE (1987) *Statistical Methods in Cancer Research. Volume II – The Design and Analysis of Cohort Studies*. IARC Scientific Publications No. 82. International Agency for Research on Cancer: Lyon

Enger SM, Ross RK, Henderson B and Bernstein L (1997) Breastfeeding history, pregnancy experience and risk of breast cancer. *Br J Cancer* **76:** 118–123

Hsieh C-C, Wuu J, Trichopoulos D, Adami H-O and Ekbom A (1999) Gender of offspring and maternal breast cancer risk. *Int J Cancer* **81:** 335–338

Kroman N, Wohlfahrt J, Andersen KW, Mouridsen HT, Westergaard T and Melbye M (1997) Time since childbirth and prognosis in primary breast cancer: population-based study. *Br Med J* **315:** 851–855

Lambe M, Hsieh C-C, Trichopoulos D, Ekbom A, Pavia M and Adami H-O (1994) Transient increase in the risk of breast cancer after giving birth. *N Engl J Med* **331:** 5–9

Melbye M, Wohlfahrt J, Olsen JH, Frisch M, Westergaard T, Helweg-Larsen K and Andersen PK (1997) Induced abortion and the risk of breast cancer. *N Engl J Med* **336:** 81–85

Storm HH (1991) *The Danish Cancer Registry, a Self-reporting National Cancer Registration System with Elements of Active Data Collection*, pp. 220–236. IARC Scientific Publication **95**

Troisi R, Weiss HA, Hoover RN, Potischman N, Swanson CA, Brogan DR, Coates RJ, Gammon MD, Malone KE, Daling JR and Brinton LA (1998) Pregnancy characteristics and maternal risk of breast cancer. *Epidemiology* **9:** 641–647

Westergaard T, Wohlfahrt J, Aaby P and Melbye M (1997) Population based study of rates of multiple pregnancies in Denmark, 1980–94. *Br Med J* **314:** 775–779

Wohlfahrt J and Melbye M (1999) Maternal risk of breast cancer and birth characteristics of offspring by time since birth. *Epidemiology* **10:** 441–444

*© 2000 Cancer Research Campaign*

